# Severe Dementia Predicts Weight Loss by the Time of Death

**DOI:** 10.3389/fneur.2021.610302

**Published:** 2021-05-14

**Authors:** Aline Maria M. Ciciliati, Izabela Ono Adriazola, Daniela Souza Farias-Itao, Carlos Augusto Pasqualucci, Renata Elaine Paraizo Leite, Ricardo Nitrini, Lea T. Grinberg, Wilson Jacob-Filho, Claudia Kimie Suemoto

**Affiliations:** ^1^Discipline of Geriatrics, University of São Paulo Medical School, São Paulo, Brazil; ^2^Department of Pathology, University of São Paulo Medical School, São Paulo, Brazil; ^3^Department of Neurology, University of São Paulo Medical School, São Paulo, Brazil; ^4^Department of Neurology and Pathology, Memory and Aging Center, University of California, San Francisco, San Francisco, CA, United States

**Keywords:** dementia, cognitive decline, body mass index, weight loss, epidemiology, aging

## Abstract

**Background:** Body mass index (BMI) in midlife is associated with dementia. However, the association between BMI and late-life obesity is controversial. Few studies have investigated the association between BMI and cognitive performance near the time of death using data from autopsy examination. We aimed to investigate the association between BMI and dementia in deceased individuals who underwent a full-body autopsy examination.

**Methods:** Weight and height were measured before the autopsy exam. Cognitive function before death was investigated using the Clinical Dementia Rating (CDR) scale. The cross-sectional association between BMI and dementia was investigated using linear regression models adjusted for sociodemographic and clinical variables.

**Results:** We included 1,090 individuals (mean age 69.5 ± 13.5 years old, 46% women). Most participants (56%) had a normal BMI (18.5–24.9 kg/m^2^), and the prevalence of dementia was 16%. Twenty-four percent of the sample had cancer, including 76 cases diagnosed only by the autopsy examination. Moderate and severe dementia were associated with lower BMI compared with participants with normal cognition in fully adjusted models (moderate: β = −1.92, 95% CI = −3.77 to −0.06, *p* = 0.042; severe: β = −2.91, 95% CI = −3.97 to −1.86, *p* < 0.001).

**Conclusion:** BMI was associated with moderate and severe dementia in late life, but we did not find associations of BMI with less advanced dementia stages.

## Introduction

Dementia affects 46 million people worldwide, and 58% of these people live in low-/middle-income countries (1). Obesity is a known risk factor for dementia, but dementia itself can also affect body weight through changes in appetite and other behavioral problems (2). Body mass index (BMI) is an easy measure to assess nutritional status. Besides, BMI has a strong correlation with total-body and visceral adiposity (3).

In several studies, overweight and obesity evaluated with BMI in midlife (between the fifth and the sixth decades of life) were linked to worse cognitive performance (4–16). However, in older individuals, BMI was not associated with higher dementia risk in some studies, and even higher BMI values were related to lower dementia risk (9, 11, 15, 17). These unexpected findings could be due to reverse causation, as dementia has an insidious onset and a long and asymptomatic preclinical phase, and the weight loss may be secondary to the cognitive and neuropsychiatric changes that occur early during the disease course (11, 12, 18). Survival bias could be another possible explanation since individuals with higher BMI may not survive until old age to develop dementia symptoms, as they suffer a higher burden of cardiovascular disease (8). Another reason for BMI to be found to be protective against dementia could be the presence of consumptive conditions (e.g., cancer), which lead to weight loss, but were not fully adjusted in previous studies (6, 7, 17, 19). Indeed, the presence of undiagnosed cancer could bias the association between BMI and adverse health outcomes (20). Therefore, we investigated the relationship between BMI near the time of death and cognitive performance in 1,090 deceased individuals submitted to a full-body autopsy, which allowed the detection of undiagnosed cancer.

## Methods

### Participants

This study used the Biobank for Aging Studies (BAS) collection from the University of São Paulo Medical School (Brazil). Data were collected at the São Paulo Autopsy Service that receives individuals who died from non-traumatic deaths and required a full-body autopsy to determine the cause of death (21). Trained nurses/gerontologists interviewed family members using a semistructured questionnaire that included sociodemographic variables, clinical history, and functional and cognitive assessments. To ensure data reliability, informants had to have at least weekly contact with the deceased (22). Exclusion criteria for the BAS are individuals with macroscopic cerebral lesions that required brain examination by the pathologist to determine the cause of death or the presence of severe acidosis (pH < 6.5) (21).

For this study, we also excluded individuals under 50 years old, those with incomplete data on weight, height, the Clinical Dementia Rating (CDR) scale, or covariates. Body weight was obtained using an electronic scale, and height was measured with a stadiometer with the deceased individuals in the supine position without clothes and shoes (23). All participants underwent a full-autopsy exam. Assessment of the death certificates was carried out to include possible cases of undiagnosed cancer during life. The local ethical committee approved the research (protocol number 04655612.9.0000.0065), and family members of the deceased signed an informed consent document.

### Cognitive Evaluation

The CDR is an extensively validated instrument, translated into several languages (24). It is based on clinical symptoms of dementia, not depending on any other psychometric test for its application. It can be applied by non-medical professionals and offers different modes of interpretation, including the possibility of comparative use of a patient's total score over the years (25). It classifies cognitive performance into six categories (memory, orientation, judgment and problem solving, community affairs, home and hobbies, and personal care) through a semistructured interview applied to the individual and an informant. By design, we only used the informant part of the CDR in this study. CDR scores range from 0 (no dementia) to 0.5 (questionable dementia), 1 (mild dementia), 2 (moderate dementia), and 3 (severe dementia) (24). The scoring system is based on individual scores of all categories. Memory was considered the primary category, and others are secondary. It is also possible to compute the total CDR sum of the boxes (CDR-SOB) score by summing the points in each of the six cognitive ability categories (26, 27). The CDR-SOB scores range from 0 to 18.

### Covariates

Possible confounders for the association of BMI and CDR were age, sex (male or female), race (White, Black, or Asian), years of education, physical inactivity (defined by <three times of physical activity per week), alcohol use (never consumed any alcohol, current or previous history of alcohol use), current smoking (never smoked, current smoker, or previous use of tobacco), and history of previous medical diagnosis of hypertension, diabetes, coronary heart disease, heart failure, and cancer, which was later compared to death certificates to identify possible non-diagnosed cases during the lifetime.

### Statistical Analysis

We compared BMI categories with categorical variables using the chi-square test and continuous variables using one-way ANOVA or the Kruskal–Wallis tests. We hypothesized that dementia presence and severity would determine weight at the time of death. Therefore, we used a linear regression in which the predictor variable was the CDR categories, and the outcome was the measured BMI (Table 1). Age, sex, race, education, hypertension, diabetes mellitus, heart failure, dyslipidemia, cancer, sedentary lifestyle, smoking, and alcohol use were considered confounding factors in the association between BMI and CDR. Besides, we performed a sensitivity analysis for this association using the CDR sum of boxes as the outcome. Moreover, since cancer and current smoking could be a strong predictor of BMI (28), we conducted an additional sensitivity analysis excluding participants with these conditions and using adjusted linear models for the same set of covariates.

**Table 1:** Sociodemographic and clinical characteristics by body mass index (BMI) categories (*n* = 1,090).

**BMI (kg/m^**2**^)**	**All**	**<18.5**	**18.5–24.9**	**25–29.9**	**>30**	***p***
	***N* = 1,090**	***N* = 163**	***N* = 569**	***N* = 241**	***N* = 117**	
Age^*^ (years),	69.5	74.5	71.0	65.5	63.3	<0.001
mean (SD)	(13.5)	(13.3)	(13.3)	(12.5)	(12)	
Male^†^, %	54.5	49.0	59.4	53.1	41.8	0.001
Race^†^, %						0.055
White	65.4	55.8	66.2	68.4	66.6	
Black	31.3	38.6	30.4	28.2	32.4	
Asian	3.2	5.5	3.1	3.3	0.8	
Education^**^ (years),	4.0	4.0	4.0	4.0	4.0	0.005
median (range)	(0–22)	(0–22)	(0–15)	(0–20)	(0–18)	
Physical inactivity^†^, %	60.0	80.3	58.3	50.6	58.9	0.572
Hypertension^†^, %	62.0	42.3	59.2	73.0	80.3	<0.001
Diabetes^†^, %	30.4	25.1	26.3	36.5	45.2	<0.001
Coronary artery disease^†^, %	19.8	6.7	18.4	28.6	26.4	<0.001
Heart failure^†^, %	17.8	6.1	17.7	21.5	27.3	<0.001
Dyslipidemia^†^, %	12.4	9.8	9.4	19.0	17.0	<0.001
Cancer^†^, %	24.4	34.3	26.0	17.8	16.2	<0.001
Smoking^†^, %						0.688
Current	29.0	27.6	30.4	30.22	22.2	
Previous	25.9	29.4	24.7	26.9	24.7	
Never	44.9	42.9	44.8	42.7	52.9	
Alcohol use^†^, %						0.018
Current	29.2	23.3	29.3	32.7	29.9	
Previous	17.6	24.5	18.1	15.7	7.6	
Never	53.1	52.1	52.3	51.4	61.5	

* *One-way ANOVA*.

** *Kruskal–Wallis test*.

† *Chi-square test*.

To explore the diversity of race/ethnicity in our sample, we investigated whether the association between BMI and cognition was different between Black and White participants. For this analysis, we excluded Asians (*n* = 35) and created an interaction term between race and CDR. In addition, we conducted stratified analyses by race for the association between BMI and CDR. Statistical analysis was performed with STATA 15 (StataCorp. 2017, College Station, TX). The level of significance was set at 0.05 in two-tailed tests.

## Results

**Figure 1:**
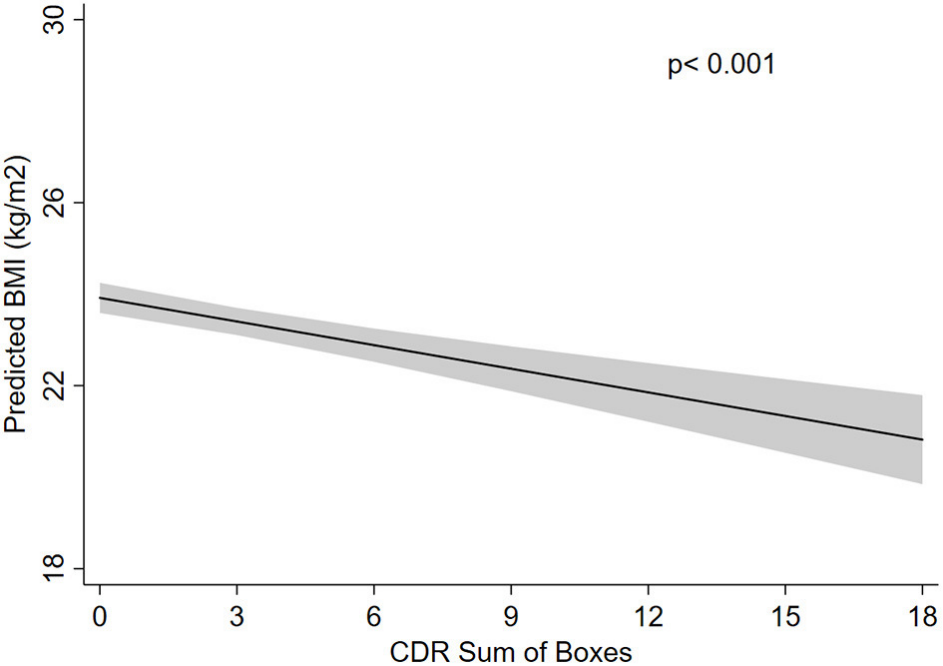
Absolute frequency of participants regarding the Clinical Dementia Rating (CDR) scores and body mass index (BMI) categories.

A total of 1,090 participants were included in this study. The final sample consisted mostly of men (55%), with a mean age of 69.5 ± 13.5 years old and median educational attainment of 4 years (range: 0–22 years). The most frequent clinical conditions were hypertension (62%), diabetes (31%), and physical inactivity (60%) (Table 1). The majority of the sample had a normal BMI (52%) with a mean BMI of 23.5 ± 5.5 kg/m^2^ (Figure 1). The prevalence of cognitive impairment (CDR ≥ 1) was 16%. Approximately 24% of individuals were diagnosed with cancer, and 76 (7%) of the cancers were detected during the autopsy exam only.

We observed that underweight was associated with older age, while obesity (BMI > 30 kg/m^2^) was more prevalent in younger individuals. Men had a lower BMI, accounting for almost 60% of malnourished individuals. Higher education level was associated with higher BMI, and Black individuals had, on average, lower BMI than White individuals (Table 1). Regarding clinical characteristics, hypertension, diabetes, coronary artery disease, heart failure, and dyslipidemia were associated with overweight and obesity (Table 1). Alcohol use was associated with lower BMI levels (Table 1).

In the unadjusted model, we did not observe BMI differences among participants with normal cognition, questionable dementia, and mild dementia, while moderate and severe dementia were associated with lower BMI (moderate dementia: β = −2.77, 95% CI = −4.73 to −0.81, *p* = 0.005; severe dementia: β = −4.47, 95% CI = −5.49 to −3.45, *p* < 0.001) (Table 2). Even after adjusting the analysis for all confounding variables, these associations remained significant. Compared with participants with normal cognition, participants with moderate dementia had on average 1.92 kg/m^2^ of BMI less than participants without dementia (β = −1.92, 95% CI = −3.77 to −0.06, *p* = 0.042). Participants with severe dementia had on average 2.91 kg/m^2^ less than those with normal cognition (β = −2.91, 95% CI = −3.97 to −1.86, *p* < 0.001) (Table 2). Association between BMI and all covariates is presented in Supplementary Table 1. Age, education, heart failure, cancer, and current alcohol use were related to BMI.

**Table 2:** Association between body mass index (BMI) and clinical dementia rating (CDR) categories (*n* = 1,090).

	**Crude**		**Model 1**		**Model 2**	
	**β (95% CI)**	***p***	**β (95% CI)**	***p***	**β (95% CI)**	***p***
CDR 0.5	−0.89 (−2.53; 0.75)	0.286	−0.41 (−2.01; 1.20)	0.620	−0.84 (−2.39; 0.71)	0.288
CDR 1	−0.65 (−2.54; 1.25)	0.502	0.40 (−1.48; 2.27)	0.678	0.04 (−1.76; 1.84)	0.965
CDR 2	−2.77 (−4.73; −0.81)	0.005	−2.02 (−3.96; −0.09)	0.039	−1.92 (−3.77; −0.06)	0.042
CDR 3	−4.47 (−5.49; −3.45)	<0.001	−3.47 (−4.52; −2.42)	<0.001	−2.91 (−3.97; −1.86)	<0.001

*Reference: CDR 0 (without dementia)*.

*CDR 0.5 (questionable dementia), CDR 1 (mild dementia), CDR 2 (moderate dementia), and CDR 3 (severe dementia)*.

*Model 1: linear regression model, adjusted for age, sex, race, and education*.

*Model 2: linear regression model, adjusted for age, sex, race, education, hypertension, diabetes, coronary artery disease, heart failure, dyslipidemia, cancer, physical inactivity, alcohol use, and smoking*.

**Figure 2:**
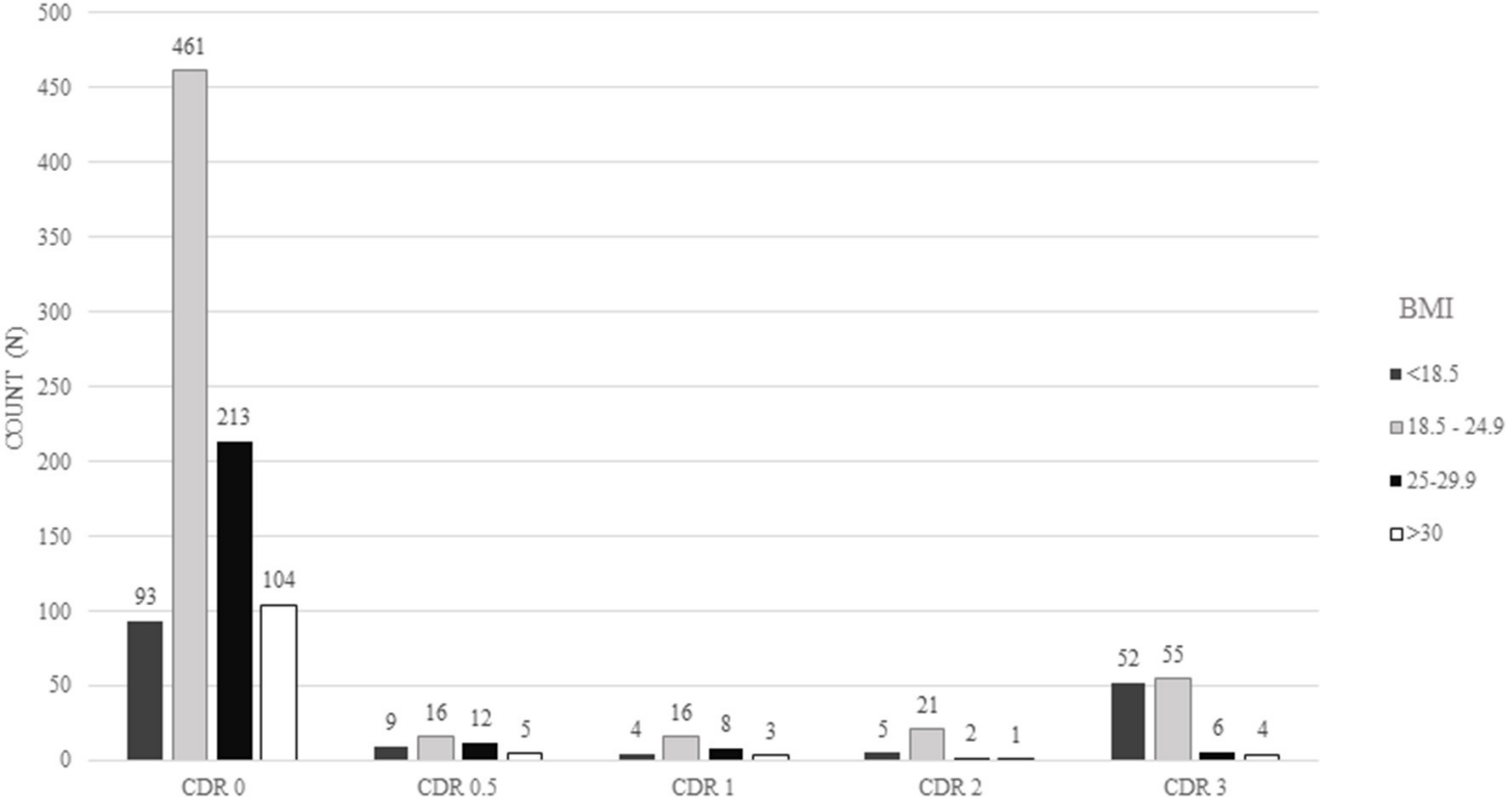
Predicted body mass index (BMI) according to Clinical Dementia Rating sum of boxes (CDR-SOB). Predicted BMI was calculated using a linear regression model adjusted for age, sex, race, education, hypertension, diabetes mellitus, heart failure, dyslipidemia, cancer, sedentary lifestyle, smoking, and alcohol use.

We performed two different sensitivity analyses that confirm our finding. We first excluded participants with a cancer diagnosis or current smoking. The exclusion of these participants did not change our results (moderate dementia: β = −2.24, 95% CI = −4.48 to 0.06, *p* = 0.044; severe dementia: β = −3.16, 95% CI = −4.52 to −1.85, *p* < 0.001) (Table 3). In another approach, we used the CDR sum of boxes as the exposure variable, and we found on average a 0.178-kg/m^2^ decrease in BMI for each 1-unit increase in the CDR sum of boxes in the fully adjusted model (β = −0.178, 95% CI = −0.238 to −0.119, *p* < 0.001) (Figure 2).

**Table 3:** Association between body mass index (BMI) and clinical dementia rating (CDR) categories, excluding participants with cancer and current smokers (*n* = 575).

	**Crude**		**Model 1**		**Model 2**	
	**β (95% CI)**	***p***	**β (95% CI)**	***p***	**β (95% CI)**	***p***
CDR 0.5	1.64 (−3.71; 0.42)	0.119	−1.18 (−3.16; 0.80)	0.242	−1.12 (−3.07; 0.83)	0.258
CDR 1	−2.60 (−5.07; 0.14)	0.039	−1.19 (−3.60; 1.22)	0.332	−1.04 (−3.40; 1.32)	0.387
CDR 2	−3.71 (−6.00; −1.42)	0.002	−2.63 (−4.84; −0.42)	0.019	−2.24 (−4.48; −0.06)	0.044
CDR 3	−5.21 (−6.48; −3.94)	<0.001	−3.95 (−5.22; −2.68)	<0.0001	−3.16 (−4.52; −1.85)	<0.0001

*Reference: CDR 0 (without dementia)*.

*CDR 0.5 (questionable dementia), CDR 1 (mild dementia), CDR 2 (moderate dementia), and CDR 3 (severe dementia)*.

*Model 1: linear regression model, adjusted for age, sex, race, and education*.

*Model 2: linear regression model, adjusted for age, sex, race, education, hypertension, diabetes, coronary artery disease, heart failure, dyslipidemia, physical inactivity, and alcohol use*.

We also investigated the interaction between race and CDR on BMI values. We did not find a significant interaction between race and CDR (*p* = 0.328). Moreover, we found similar associations between CDR and BMI in White and Black participants in stratified analyses by race, with lower BMI values among participants with severe dementia compared with participants with normal cognition (Supplementary Table 2).

## Discussion

We found an association of BMI with moderate and severe dementia, suggesting that individuals with more advanced dementia are more likely to have lower body weight by the time of death. We did not find associations between BMI and early dementia stages. When we excluded individuals with cancer and current smokers, moderate and severe dementia were still associated with lower BMI. Moreover, we did not find evidence of interaction between race and cognitive status on BMI.

The relationship between BMI and dementia varies along the life course. In middle-aged adults, obesity was linked to brain atrophy (29), worse cognitive performance, and higher mortality (30, 31). This association may be explained by diverse pathophysiological pathways, including systemic and central nervous system inflammation (32), triggered by the production of hormones like leptin and other inflammatory cytokines (e.g., tumor necrosis factor-α and interleukins) by the adipose tissue. After long-term exposure, inflammation is believed to damage cerebral arteries, raising the risk for vascular dementia and Alzheimer's disease (30, 33–39). In animal models, intestinal microbiota dysbiosis linked to obesity also stimulates the neuroinflammatory response, but human data regarding this hypothesis are still lacking (35).

On the other hand, obesity was not associated with higher dementia risk in late life, while lower BMI was related to higher dementia risk (8, 9, 11, 15). Along with these findings, we found that moderate and severe dementia were related to lower BMI. One possible explanation for the lower BMI to be related to dementia could be reverse causation. Even decades before the dementia diagnosis, deposition of beta-amyloid and tau proteins in the olfactory bulb leads to impaired smelling capacity. In addition, the deposition of beta-amyloid protein on the hypothalamic nuclei is among the earliest neuropathological changes in Alzheimer's disease (36). The impaired smell sensibility can impact food tasting, decreased calorie intake, and lead to weight loss (37, 38). Deposition of beta-amyloid proteins in areas of the brain responsible for energy and hunger regulation can also decrease hormones, like leptin, cholecystokinin, and serotonin. Phosphorylated tau proteins are also linked to the avoidance of high-fat diets, related to higher BMI (39). However, we did not find that early dementia stages were related to lower BMI, only moderate and advanced dementia were associated with lower BMI values. These findings could be caused by neuropsychiatric symptoms that are common among individuals with more advanced dementia stages. Depressive symptoms often include appetite loss and apathy and the absence of motivation or initiative to obtain food and eat (18). Besides, the disturbance of neurons that produce neurotransmitters (e.g., norepinephrine) may result in anxiety and appetite dysfunction (38). Besides, patients with moderate and advanced dementia are at least partially dependent on basic activities of daily life, such as obtaining and cooking food (19). Another crucial factor to consider is dysphagia. A systematic review showed that 57% of patients with advanced dementia had dysphagia, which is an important cause of weight loss, malnutrition, dehydration, and additional functional loss (40).

A fundamental aspect of our study is that we could include data from the autopsy examination, while most studies on the association between BMI and dementia used clinical data (8, 9, 12). We were able to detect 76 additional cancer cases, which increased the frequency of neoplasms from 20 to 24% in our sample. The investigation of consumptive diseases like cancer is of paramount importance when studying the effect of BMI on health outcomes. Cancer is associated with severe inflammation and cachexia syndrome that lead to weight loss (41–44). Another strength of our study is that the data were collected at the São Paulo Autopsy Service, a general autopsy service. Therefore, most participants do not have cognitive impairment or have mild symptoms of dementia, which more accurately represent community-dwelling adults regarding dementia prevalence and severity. Another advantage is that this study was conducted in a sample with diverse races compared with other studies based on North American, European, and Asian populations (8, 9, 11, 14). Dementia risk seems to be higher in African Americans compared with non-Hispanic Whites (45). Since Brazil is a multiracial country (46), we were able to explore whether the association between BMI and cognition differed by race. Severe dementia was related to lower BMI in both White and Black participants, but we did not find an effect modification by race in this association.

Nonetheless, our study needs to be considered in light of its limitations. The cross-sectional design does not allow establishing cause–effect relationships. Moreover, despite extensive literature showing a high correlation between BMI and body adiposity (41), we did not directly measure body adipose composition. Instead, we used BMI, which is easy to perform in clinical settings and is correlated with body adiposity (3). Finally, we do not have available information on the neuropathological examination of these cases at this moment.

Our main finding was that individuals with moderate and severe stages of dementia had lower BMI, in agreement with previous studies (4–12, 16). However, no significant association was found with earlier dementia stages. Our study contributes to understanding the association between BMI and dementia at the end of life, including the information of undiagnosed cancer during life that could have contributed to weight loss unrelated to dementia. Further studies are needed to establish the pathophysiology and mechanisms directly leading to weight loss from preclinical to advanced dementia.

## Data Availability Statement

The raw data supporting the conclusions of this article will be made available by the authors, without undue reservation.

## Ethics Statement

The studies involving human participants were reviewed and approved by University of São Paulo's ethical committee. The patients/participants provided their written informed consent to participate in this study.

## Author Contributions

AC: Conception and design, data collection, data analysis, drafting, revision and final approval of the version to be published. IA: Conception and design, drafting, revision and final approval of the version to be published. DS, CP, RL, RN, and LG: Revision and final approval of the version to be published. WJ-F: Conception and design, data collection, data analysis, drafting, revision and final approval of the version to be published. CS: Conception and design, data analysis, revision and final approval of the version to be published. All authors contributed to the article and approved the submitted version.

## Conflict of Interest

The authors declare that the research was conducted in the absence of any commercial or financial relationships that could be construed as a potential conflict of interest.
