# Supplementary material for: Severe Dementia Predicts Weight Loss by the Time of Death
Source: Front Neurol. 2021 May 14;12:610302. doi: 10.3389/fneur.2021.610302 (PMC8160379; doi:10.3389/fneur.2021.610302)
Supplement: Supplementary file 1 [file Data_Sheet_1.docx]

# Supplementary data

**Supplementary Table 1.** Association of cognitive status and all covariates with body mass index (n=1,090)

|  | **β**  **(95%CI)** | **p** |
| --- | --- | --- |
| **CDR 0.5** | -0.84 (-2.39; 0.71) | 0.288 |
| **CDR 1** | 0.04 (-1.76; 1.84) | 0.965 |
| **CDR 2** | -1.92 (-3.77; -0.06) | 0.042 |
| **CDR 3** | -2.91 (-3.97; -1.86) | <0.001 |
| **AGE** | -0.09 (-0.12; -0.06) | <0.001 |
| **MALE** | -0.44 (-1.12; 0.23) | 0.202 |
| **RACE** |  |  |
| *BLACK* | -0.65 (-1.30; 0.00) | 0.050 |
| *ASIAN* | -1.28 (-3.03; 0.45) | 0.148 |
| **EDUCATION** | 0.09 (0.01; 0.17) | 0.027 |
| **PHYSICAL INACTIVITY** | -0.19 (0.82; 0.43) | 0.543 |
| **HYPERTENSION** | 1.71 (1.05; 2.37) | <0.0001 |
| **DIABETES** | 0.87 (0.20; 1.55) | 0.011 |
| **CORONARY ARTERY DISEASE** | 0.66 (0.11; 1.44) | 0.095 |
| **HEART FAILURE** | 1.01 (0.21; 1.82) | 0.013 |
| **DYSLIPIDEMIA** | 0.95 (0.03; 1.87) | 0.042 |
| **CANCER** | -1.23 (-1.93; -0.53) | <0.0001 |
| **SMOKING** |  |  |
| *CURRENT* | 0.21 (0.56; 0.99) | 0.596 |
| *PREVIOUS* | 1.95 (1.01; 4.92) | 0.196 |
| *NEVER* | -0.68 (-1.47; 0.09) | 0.086 |
| **ALCOHOL USE** |  |  |
| *CURRENT* | -1.55 (-2.69; -0.41) | 0.008 |
| *PREVIOUS* | -1.07 (-1.99; -0.16) | 0.021 |
| *NEVER* | 0.12 (-0.73; 0.99) | 0.767 |

Linear regression model including all covariates in the table.

**Supplementary Table 2.** Association between body mass index (BMI) and Clinical Dementia Rating (CDR) categories stratified by race (n=1,055)

|  | White |  | Black |  |
| --- | --- | --- | --- | --- |
|  | **N=713** |  | **N=342** |  |
|  | **β (95%CI)** | **p** | **β (95%CI)** | **p** |
| CDR=0.5 | -1.48 (-3.15; 0.20) | 0.084 | 1.32 (-2.35; 5.00) | 0.478 |
| CDR=1 | -1.26 (-3.86; 1.34) | 0.342 | 1.56 (-1.26; 4.38) | 0.277 |
| CDR=2 | -1.48 (-3.85; 0.89) | 0.221 | -2.77 (-5.95; 0.40) | 0.087 |
| CDR=3 | -3.14 (-4.34; -1.94) | <0.001 | -2.21 (-4.40; -0.03) | 0.047 |

Reference: CDR 0 (without dementia)

CDR 0.5 (questionable dementia), CDR 1 (mild dementia), CDR 2 (moderate dementia), and CDR 3 (severe dementia)

Linear regression model, adjusted for age, sex, race, education, hypertension, diabetes, coronary artery disease, heart failure, dyslipidemia, cancer, physical inactivity, alcohol use, smoking.
